# Computational Investigations on Reaction Mechanisms of the Covalent Inhibitors Ponatinib and Analogs Targeting the Extracellular Signal-Regulated Kinases

**DOI:** 10.3390/ijms242015223

**Published:** 2023-10-16

**Authors:** Yafeng Tian, Mi Zhang, Panpan Heng, Hua Hou, Baoshan Wang

**Affiliations:** College of Chemistry and Molecular Sciences, Wuhan University, Wuhan 430072, China; yafeng@whu.edu.cn (Y.T.); zhangmi@whu.edu.cn (M.Z.); hengpanpan@whu.edu.cn (P.H.); houhua@whu.edu.cn (H.H.)

**Keywords:** ERK regulation, covalent inhibitors, thiol addition reaction, quantum chemical calculation, molecular dynamics simulations

## Abstract

As an important cancer therapeutic target, extracellular signal-regulated kinases (ERK) are involved in triggering various cellular responses in tumors. Regulation of the ERK signaling pathway by the small molecular inhibitors is highly desired for the sake of cancer therapy. In contrast to the routine inhibitors targeting ERKs through long-range non-bonding interactions, Ponatinib, a covalent inhibitor to ERK2 with a macrocyclic structure characterized by the α,β-C=C unsaturated ketone, can form the stable -C(S)-C(H)-type complex via the four-center barrier due to the nucleophilic addition reaction of the thiol group of the Cys166 residue of ERK2 with the C=C double bond of Ponatinib with reaction free-energy barrier of 47.2 kcal/mol. Reaction mechanisms for the covalent binding were calculated using QM/MM methods and molecular dynamics simulations. The interaction modes and the corresponding binding free energies were obtained for the non-covalent and covalent complexation. The binding free energies of the non-covalent and covalent inhibitions are 14.8 kcal/mol and 33.4 kcal/mol, respectively. The mechanistic study stimulated a rational design on the modified Ponatinib structure by substituting the C=C bond with the C=N bond. It was demonstrated that the new compound exhibits better inhibition activity toward ERK2 in term of both thermodynamic and kinetic aspects through the covalent binding with a lower reaction free-energy barrier of 23.1 kcal/mol. The present theoretical work sheds new light on the development of the covalent inhibitors for the regulation of ERKs.

## 1. Introduction

Extracellular signal-regulated kinases (ERKs) are a class of protein kinases belonging to the mitogen-activated protein kinases (MAPK) family. These kinases are critical components to the RAS/RAF/MEK (mitogen-activated protein kinase kinase)/ERK pathway and are involved in triggering various cellular responses in tumors, such as cell survival, differentiation, and progression [1,2]. It has been demonstrated that the ERKs act as the “switch” in the downstream of the signaling pathway, inducing the development of cancer triggered by various growth factors or mutant proteins [3]. For the cancers like ovarian cancer, bladder cancer, lung cancer, and breast cancer, the ERK subtypes, namely ERK1 and ERK2, were found to be amplified significantly. Moreover, the overactivation of ERKs is responsible for the upregulation of anti-apoptotic proteins, leading to drug resistance in various types of cancers [4,5,6,7]. Although several small molecular inhibitors targeted on BRAF and MEK such as Dabrafenib and Vemurafenib have passed clinical validation in treatment of BRAF-mutant melanomas [8,9], several studies have demonstrated that drug resistance occurred, and ERK 1/2 can be re-activated after the prolonged administration of inhibitors of receptor tyrosine kinases (RTK), RAF, and MEK [10,11,12]. In comparison to the upstream kinases, few mutations are observed in cancer cell genomes due to their conservative amino acid sequence and structure [13]. It has been demonstrated that the inhibitors targeted straightforwardly on ERK 1/2 exhibited a higher specificity and lower risk of drug resistance compared with RAF or MEK inhibitors [14]. Therefore, ERK is believed to be an important cancer therapeutic target, and the regulation of the ERK signaling pathway has become a hot topic among researchers.

Over the past few decades, several classes of inhibitors targeting ERKs, for instance ATP competitive inhibitors and ERK 1/2 allosteric inhibitors [15,16], have been suggested for the sake of cancer therapy. However, these inhibitors are still in either laboratory development or clinical trial stages. In recent years, more and more targeted covalent inhibitors (TCIs) have been approved for the treatment of cancers and other diseases by the Food and Drug Administration (FDA). TCIs are also treated as one of the effective strategies to inhibit ERK via the covalent bond with the residue cysteine 166 (Cys166) of ERK by means of the nucleophilic addition reaction [17,18,19,20,21,22,23]. In contrast to the traditional inhibitors, the covalent inhibitors refer to the small molecules that are capable of binding covalently to the target [24]. Regarding to the biological functions, the small molecules associate reversibly with the target enzyme through non-bonding interactions (e.g., hydrogen bond, van der Waals interaction, hydrophobic interaction, etc.) between the reactive moiety of the inhibitor and the close proximity of the reactive residue of the targeted enzyme. Subsequently, the reactive center, i.e., warhead, of the inhibitor, which is usually an electrophilic functional group, forms the covalent bond to the reactive entity of the enzyme [25]. Interestingly, the earliest covalent inhibitor can be traced back to acetylsalicylic acid, commonly known as aspirin. Until 1970s, aspirin was discovered to irreversibly inhibit acetylation of cyclooxygenase by covalently bonding to the serine residue [26,27]. Recently, several covalent inhibitors have been approved for clinic use. For instance, the drug nirmatrelvir, targeting the main protease of severe acute respiratory syndrome coronavirus 2 (e.g., SARS-CoV-2 Mpro), has been issued emergency-use authorization for medication [28,29]. Due to the nature of the covalent bonding, the covalent inhibitors have a more persistent effect than the non-covalent inhibitors. Moreover, the covalent inhibitors exhibit a high level of specificity to the target proteins, reducing the impact on other proteins to lower drug resistance. However, the computational design of the covalent inhibitor is challenging because the chemical reactions are involved rather than the non-bonding interactions. Moreover, unique challenges, including the reactivity of nucleophilic residue of interest, occurrence of covalent binding in reality, and the on- and off-target selectivity of the compound, are obstacles to the discovery of covalent inhibitors [30,31,32]. Therefore, mechanistic study on the covalent inhibitors is of great significance for the development of new drugs.

**Figure 1:**
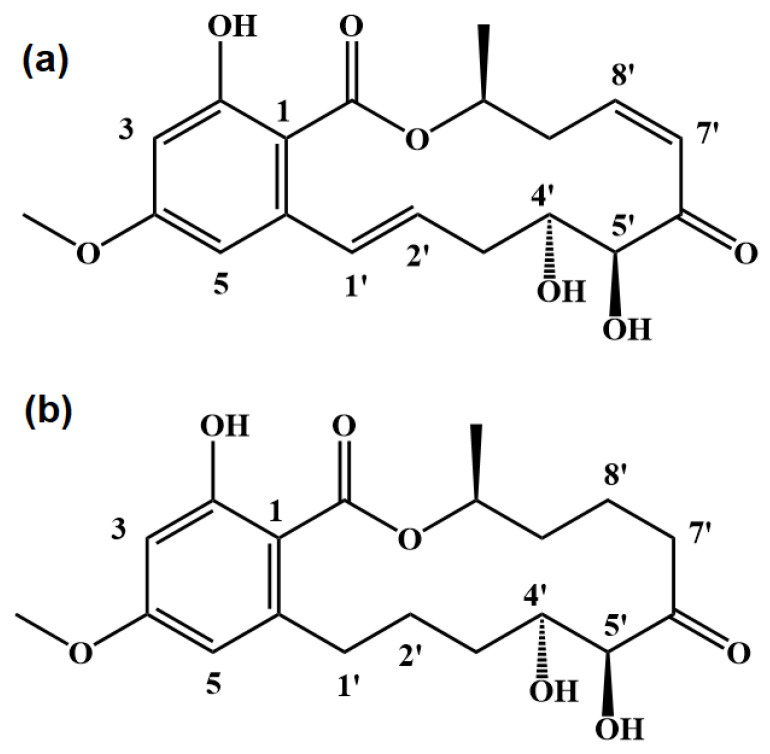
Molecular structures of Ponatinib (**a**) and the analog FR (**b**).

Ponatinib is a covalent inhibitor to ERK2, as shown in Figure 1. It appears to be a macrocyclic compound with the prototypical α,β-unsaturated ketone structure, associated with the hydrophobic benzene, methoxy, and ester groups. Two C=C double bonds are located at 1′-2′ and 7′-8′ sites, respectively. It was found that the molecular structure of Ponatinib interacts with ERK2 via the covalent bonding between the α,β-unsaturated ketone group and the cysteine residue (Cys166) for the S-C bond, leading to the strong inhibition of ERK2 activation, as indicated by the IC50 of 0.08 µM [33]. Although the molecular recognition of Ponatinib to ERK2 provided a new therapeutic approach for the treatment of malignant tumors, the corresponding structure of the Ponatinib–ERK2 complex is still elusive because the observed S-C bond distance is as long as 2.35 Å (PDB: 3W55), which is about 0.5 longer than the normally covalent S-C bond. Apparently, it is hard to distinguish whether covalent bonding has occurred between Ponatinib and the Cys166 residue of ERK2, whereas the 7′C=8′C double bond of Ponatinib is converted to a single bond, as indicated by the distance of 1.5 Å between 7′C and 8′C in the co-crystal structure. Presumably, several unsaturated sites of Ponatinib could be the possible reactive centers for the nucleophilic additions to the Cys166 residue. Therefore, it is necessary to figure out how Ponatinib covalently binds to ERK in order to provide the exact interaction modes for the Ponatinib–ERK2 complexation. It is interesting to note that the compound FR (Figure 1), being analogous to Ponatinib in structure, exhibits very low inhibitory activity against ERK2. With the saturated C-C single bonds at 1′-2′ and 7′-8′ sites, the IC50 of FR is elevated by about two orders of magnitude due to the absence of the covalent binding in the association of FR with ERK2. Since more than one reactive site exists in Ponatinib towards the Cys166 residue, the detailed reaction mechanism for the reaction of Ponatinib with ERK2 is unclear. In this work, molecular dynamics simulations were carried out to clarify the interaction mechanisms of Ponatinib with ERK2 in terms of both covalent and non-covalent binding by means of the free-energy calculations. The energetic routes for the reactions of Ponatinib with ERK2 were calculated using density functional theory. The bonding nature for the Ponatinib–ERK2 covalent interaction was revealed. The present theoretical findings offer significant insights for the rational design of the α,β-unsaturated ketone-based ERK2 covalent inhibitors. Moreover, a new covalent inhibitor with greater biological activity than Ponatinib was proposed as the α,β-C=C bond that can be substituted by the C=N double bond.

## 2. Results and Discussion

### 2.1. Noncovalent Inhibitions

**Figure 2:**
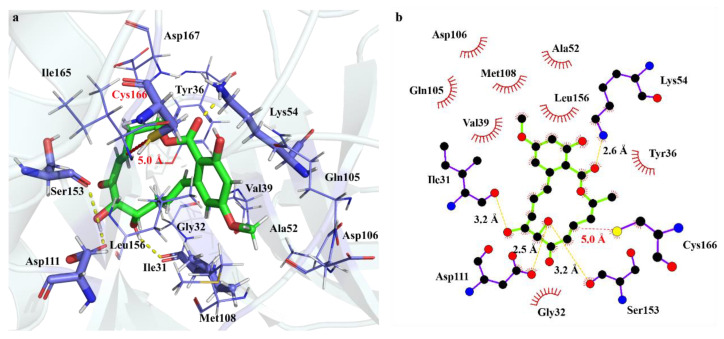
Interaction modes of Ponatinib toward ERK2 at the non-covalent binding state, (**a**) 3D diagram of the binding pocket, and (**b**) 2D interaction diagram showing the hydrogen bonds and hydrophobic residues. The green sticks represents molecule Ponatinib, blue sticks represents residues of ERK2 formed interaction with Ponatinib, yellow dash represents hydrogen bonds.

The interaction mode between Ponatinib and ERK2 at the noncovalent binding state is illustrated in Figure 2. Apparently, the interaction between the α,β-unsaturated ketone moiety and the Cys166 residue is fairly weak, as indicated by the long distance (e.g., 5.0 Å) between the 7′-C=8′-C double bond and the S atom of Cys166. Moreover, no apparent interaction of the carbonyl group with ERK2 was observed during the simulations. The dominant interaction between Ponatinib and ERK2 consists of a groups of hydrogen bonds. For instance, the hydroxyl group at 5′-C site of Ponatinib serves as a proton donor, forming two hydrogen bonds with the residues Asp111 and Ser153 of ERK2, respectively. The hydroxyl group at 4′-C of Ponatinib also acts as a proton donor in the hydrogen bond interaction with respect to the residue Ile31. Moreover, the carbonyl group of the ester moiety at 12′-C forms a C=O…H hydrogen bond with the residue Lys54. The methoxy group and the benzene ring of Ponatinib is oriented towards the β-fold region of ERK2, i.e., Val101–Met108. As a result, Ponatinib engages in the significant hydrophobic interactions with the surrounding eight residues such as Val39, Met108, Leu156, and so on.

**Figure 3:**
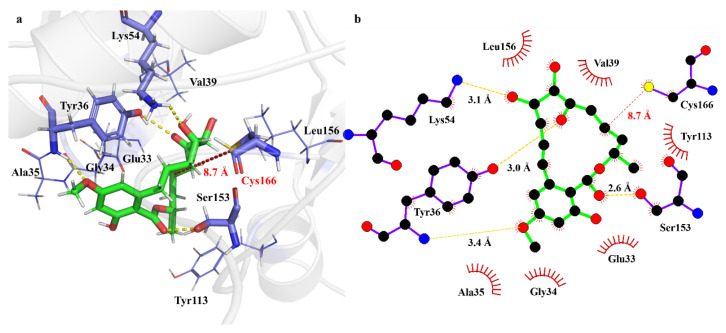
Interaction modes of FR toward ERK2 at the non-covalent binding state, (**a**) 3D diagram of the binding pocket, and (**b**) 2D interaction diagram showing the hydrogen bonds and hydrophobic residues. The green sticks represents molecule FR, blue sticks represents residues of ERK2 formed interaction with FR, yellow dash represents hydrogen bonds.

The interaction modes between FR and ERK2 are illustrated in Figure 3. Although FR can be docked into the same binding pocket as Ponatinib, it adopts an opposite orientation regarding the binding sites. Rather than the β-fold region, the terminal methoxy group is oriented towards the α-helix region of ERK2, i.e., His61–Phe78. Therefore, the hydrophobic pocket consists of only six residues, namely Glu33, Gly34, Ala35, and Tyr113 together with Leu156 and Val39, which are residues in the Ponatinib pocket. In addition, because of the presence of the saturated alkane sketches, FR stays further far away from the active Cys166 residue, as indicated by the 8′-C–S distance of 8.7 Å, excluding any chemical bonding between FR and ERK2. However, a hydrogen bond exists between the carbonyl group of ketone and the Tyr36 residue. The other significant hydrogen bond appears to be the carbonyl group of ester with respect to the Ser153 residue with the O…O distance of 2.6 Å. In contrast to Ponatinib, only the OH group at the 4′-C site forms a weak hydrogen bond with Lys54. One NH…O hydrogen bond exists between the O of the methoxy group and the Tyr36 residue.

The difference in the binding modes of Ponatinib–ERK2 from FR–ERK2 might be attributed to the significant difference in the respective inhibitory activity toward ERK2. The binding free energies of Ponatinib and FR interacting with ERK2 were predicted theoretically according to the molecular dynamics (MD) trajectories and are summarized in Table 1. For the sake of validation, the co-crystallized ligand with ERK2 (PDB: 3w55) was extracted and then re-docked into the binding pocket. The comparison between the experimental structure and the re-docked structure is shown in Figure S1 in the Supporting Information. The root mean square deviations (RMSD) between the experimental and the docked conformations was as low as 0.11 Å, proving the credibility of docking procedure. As confirmed by the 100-ns RMSD and the total energy (E_TOT_) profiles of the complexes (Figure S2 in the Supporting Information), the simulated systems appear to be stable enough in terms of both structures and energetics to reach the thermodynamic equilibrium. As could be seen in Table 1, the binding free energy of Ponatinib–ERK2 was calculated to be −14.8 kcal/mol, whereas that of FR–ERK2 is as high as 24.0 kcal/mol, as indicative of the non-spontaneous affinition for FR to ERK2, which is in agreement with the lesser activity of FR toward ERK2. In view of the individual contributions to the free energy, the significant difference between nonPE and nonFE could be attributed to the weaker hydrogen bond interactions (−37.1 kcal/mol vs. −28.4 kcal/mol) and the stronger polar solvation energy (50.7 kcal/mol vs. 75.6 kcal/mol) of the latter, as demonstrated in the respective interaction modes. The electrostatic interaction and nonpolar solvation free energies do not show any difference for nonPE and nonFE in the consideration of the theoretical uncertainty. Therefore, the α,β-unsaturated C=C bond is critical for the inhibition activity of the Ponatinib-type compounds.

### 2.2. Covalent Binding of Ponatinib to ERK2

**Figure 4:**
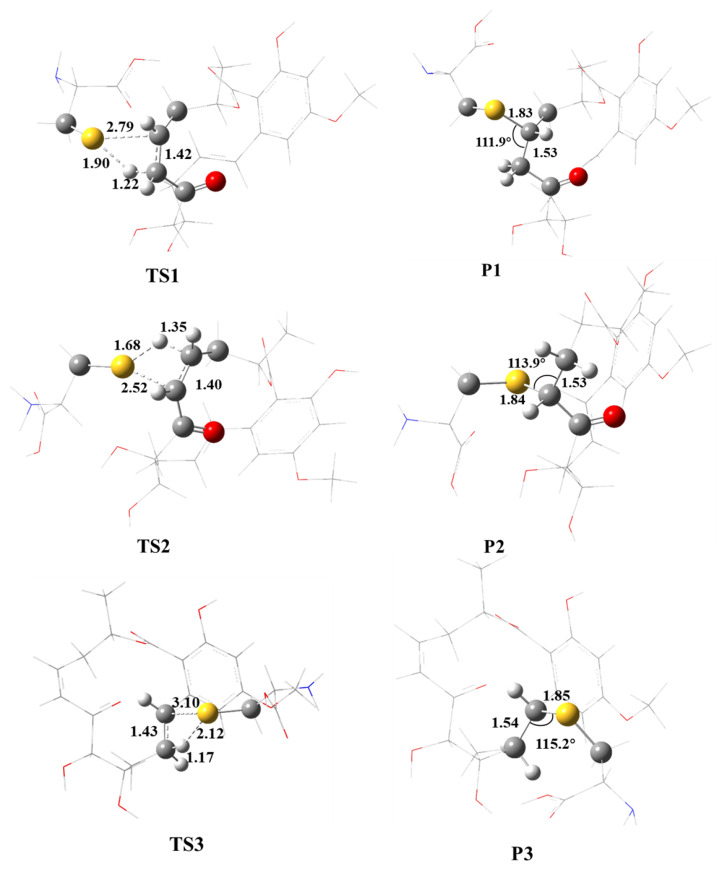
M06-2X/6-31G(d,p) optimized geometries of the transition states (TSn) and adduct (Pn) involved in the reaction of Ponatinib with cysteine. The reaction centers are shown in ball-stick models, and the rest of the atoms are in sticks. Grey balls represent C atoms; yellow balls represent S atoms; red balls represent O atoms; white balls represent H atoms. Bond distances are in Å, and angles are in degrees.

It is worth noting that the Cys166 residue of ERK2 can react with Ponatinib by the 1,2-additions to either C=C or C=O bonds. In view of the molecular structure of Ponatinib (Figure 1), four reaction centers are available, namely the C=C bonds at 1′-2′ and 7′-8′ and the C=O bonds at 6′-C and 12′-C sites. Preliminary calculations showed that the addition of the S-H bond of Cys166 to the C=O bond surmounts significant barriers, and the products are highly endothermic. Therefore, the thiol group of Cys166 prefers to react with the C=C bonds of Ponatinib. The optimized geometries of the transition states and products for the associations of S atom of Cys166 with 7′-C, 8′-C, and 1′-C are shown in Figure 4. Note that the addition of S to 2′-C is forbidden because of the steric hindrance of the macro cycle. The energetics for the respective barrier heights and reaction heats in terms of both internal and free energies are listed in Table 2. Energetically, the S atom prefers addition to the 8′-C atom to form the S-C bond via a four-center barrier TS1. The breaking S-H bond is stretched from 1.35 Å to 1.90 Å. The forming S-C and the H-C bonds are 2.79 Å and 1.22 Å, respectively. Meanwhile, the C=C double bond is elongated to 1.42 Å to form the C-C single bond in the adduct P1. The S-C bond in the adduct is 1.83 Å, which is roughly 1 Å shorter than that in TS1, implying that TS1 is an early barrier. In terms of free energy at 298.15 K, the barrier height is 47.2 kcal/mol, and the formation of the adduct P1 is exothermic by 12.1 kcal/mol. Therefore, the covalent bonding between Ponatinib and ERK2 via the Cyc166 residue can take place spontaneously and with a moderate rate.

For comparison, the addition of the S atom to 7′-C site, which is adjacent to the carbonyl, proceeds via TS2. Although the S-C bond in the adduct P2 is similar to that in P1, the reacting bonds in TS2 do show differences from those in TS1. For instance, the breaking S-H bond is 1.68 Å, which is 0.22 Å shorter than that in TS1. Moreover, the forming S-C and H-C bonds are 0.27 Å shorter and 0.14 Å longer, respectively. As a result, the barrier height for TS2 is about 9 kcal/mol higher than that for TS1, but the formation of the adduct P2 becomes less exothermic. The addition of S to the 1′-C site involves a moderate barrier TS3. The forming S-C bond is as long as 3.10 Å. In contrast, the forming H-C bond is only 1.17 Å, which is nearly the equilibrium length of the H-C bond in the adduct P3, as the breaking S-H bond is stretched to 2.12 Å. Energetically, the barrier height for TS3 is 4.4 kcal/mol higher than that for TS1, while the adduct P3 is about 4 kcal/mol less exothermic. Therefore, neither addition to 1′-C nor 7′-C could be competitive. The formation of the 8′-C-S bond should be the predominant reaction mechanism for the covalent bonding of Ponatinib to ERK2. Although the experimental S-C bond length in the Ponatinib–ERK2 complex (PDB:3W55) is apparently longer than the predicted 1.83 Å in the adduct P1, the covalent bonding to the 8′-C site is confirmed both theoretically and experimentally.

**Figure 5:**
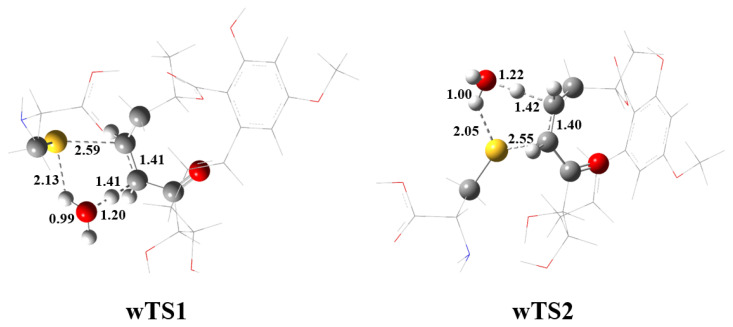
M06-2X/6-31G(d,p) optimized geometries of the H_2_O-catalyzed transition states (wTSn) involved in the reaction of Ponatinib with cysteine. The reaction centers are shown in ball-stick models, and the rest of the atoms are in sticks. Grey balls represent C atoms; yellow balls represent S atoms; red balls represent O atoms; white balls represent H atoms. Bond distances are in Å.

In the consideration of the significant barrier for TS1, a water-catalyzed mechanism for the addition of Ponatinib with cysteine was considered for completeness. It has been well established that the single water molecule is capable of reducing the barrier for the proton-transfer process considerably besides the solvent effect. The optimized H_2_O-catalyzed wTS1 is shown in Figure 5. Evidently, the H_2_O molecule acts as a bridge for the proton transfer from S to 8′-C site. The breaking S-H bond is stretched to 2.13 Å. The bridging O-H bonds are 0.99 Å and 1.20 Å, respectively. The forming H-C bond is 1.41 Å, which is about 0.2 Å longer than that in the non-catalyzed TS1. The barrier height is lowered by 13.0 kcal/mol due to the catalysis of one H_2_O molecule. The other reaction path could be catalyzed as well by one H_2_O molecule, but the barrier height for wTS2 is still higher than TS1 by about 9 kcal/mol (Figure 5). It should be noted that the H_2_O-catalyzed free-energy barrier is almost identical to the non-catalysis for both TS1 and TS2. Therefore, the addition of cysteine to Ponatinib proceeds preferentially to form the S-C bond at the 8′-C site. By means of the QM/MM method, the covalent binding energy of Ponatinib to ERK2 was calculated to be 33.4 kcal/mol, which is approximately twice that of the non-covalent binding, as indicative of the critical role of the addition reaction of Ponatinib with the Cys166 residue.

**Figure 6:**
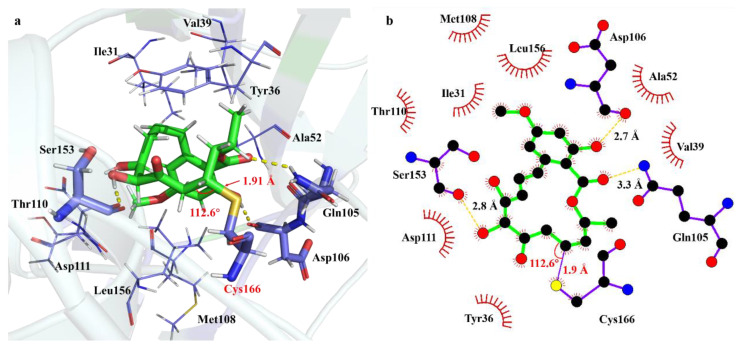
Interaction modes of Ponatinib toward ERK2 at the covalent binding state, (**a**) 3D diagram of the binding pocket, and (**b**) 2D interaction diagram showing the hydrogen bonds and hydrophobic residues. The green sticks represents molecule Ponatinib, blue sticks represents residues of ERK2 formed interaction with Ponatinib, yellow dash represents hydrogen bonds.

The covalent binding of Ponatinib with ERK2 was simulated to reveal the difference in the interaction modes from the non-covalent binding. The results are illustrated in Figure 6. Note that the covalent bond distance S-C and angle S-C-C were monitored over the 100 ns MD trajectory (Figure S3 in the Supporting Information). It appears that the covalent moiety of Ponatinib and ERK2 is fairly stable with the averaged bond length of 1.90 Å and angle of 110.9°, as confirmed by the marginal fluctuations of the temporal profiles.

As could be seen in Figure 6, although Ponatinib is still located in the same binding pocket, its conformation has changed considerably due to the existence of the covalent S-C and H-C bonds and the conversion of the trans-orientated C=C double bond to the C-C single bond. As for the hydrogen-bonding interactions, the 5′-OH with the Ser153 residue remains, but the hydrogen bond distance is shorted from 3.2 Å in nonPE to 2.8 Å in covPE. The strong hydrogen bond due to Lys54 with the ester carbonyl group disappears. Two residues, i.e., Gln105 and Asp106, migrate from the hydrophobic regions in nonPE to the O atom in the ester carbonyl group and the phenol group, respectively, to form two new hydrogen bonds. Meanwhile, two hydrogen-bonding residues in nonPE, namely Ile31 and Asp111, are moved to the hydrophobic region. The hydrophobic interaction due to the Gly32 residue is replacement by the Thr110 residue to keep the same size of the hydrophobic cavity. Above all, the covalent inhibitor Ponatinib is capable of binding to ERK2 stably in both A noncovalent state and covalent state. Both binding modes make a contribution to the inhibitory effect against ERK2 of Ponatinib. The process of the covalent bond formation is undertaken via a successive approaching path between the α,β-unsaturated ketone of Ponatinib and Cys166 of ERK2, which might lead to the fairly long S-C bond, as observed in the experimental crystal structure.

### 2.3. The Modified Ponatinib Covalent Inhibitor

As mentioned above, the reaction of Ponatinib with cysteine surmounts a barrier of 47.2 kcal/mol. Despite the H_2_O catalysis, the barrier for the S-H addition to the C=C bond is too high to be feasible because of the inherent inert nature of the C=C double bond to the weak nucleophilic addition. Therefore, it is proposed that the C=C bond could be replaced by the C=N bond, which is more reactive toward the SH group due to the strong proton affinity of the N atom.

**Figure 7:**
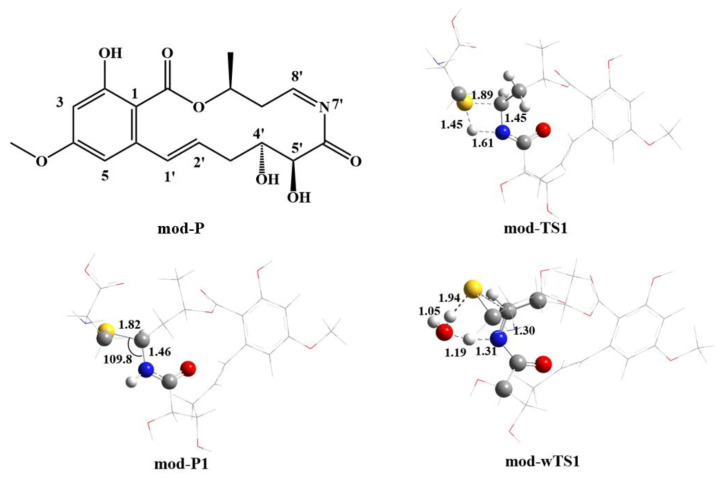
M06-2X/6-31G(d,p) optimized geometries of the H_2_O-catalyzed transition states (wTSn) involved in the reaction of Ponatinib with cysteine. The reaction centers are shown in ball-stick models, and the rest of the atoms are in sticks. Grey balls represent C atoms; yellow balls represent S atoms; red balls represent O atoms; white balls represent H atoms; blue balls represent N atoms. Bond distances are in Å, and angles are in degrees.

The modified Ponatinib is shown schematically as mod-P in Figure 7, together with the optimized transition state (mod-TS1) for the reaction of mod-P with cysteine. Evidently, mod-P is very similar to Ponatinib in geometry. The cyclic conformation of mod-P is nearly identical to that of Ponatinib, which is essential for the occurrence of mod-P in the same binding pocket of ERK2 as Ponatinib. The barrier height for the formation of the covalent -SC-NH- structure was calculated to be 37.6 kcal/mol (see Table 2), which is about 10 kcal/mol lower than that for Ponatinib. In view of the geometrical parameters, the breaking S-H bond is elongated by only 0.1 Å, i.e., 1.45 Å vs. 1.90 Å for Ponatinib. Meanwhile, the forming S-C bond is 1.89 Å, which is about 0.9 Å shorter than that for Ponatinib. The forming H-N bond is still far away (e.g., 1.61 Å) from the equilibrium length in the adduct. Taking advantage of the N substitution, the adduct between mod-P and cysteine, namely mod-P1 in Figure 7, becomes more stable by about 4 kcal/mol than that for Ponatinib. The S-C bond length does not change in the covalent bonding of mod-P to cysteine. It is interesting to note that the water molecules might play an important role in the reaction of mod-P with cysteine. As shown in Table 2, the H_2_O-catalyzed barrier mod-wTS1 is only 23.1 kcal/mol in terms of free energy, implying that the covalent bonding of mod-P can occur more feasibly than in Ponatinib in both thermodynamic and kinetic aspects. Therefore, the α,β-C=N-unsaturated ketone molecule appears to be a potential covalent inhibitor of ERK2 with better performance than Ponatinib.

**Figure 8:**
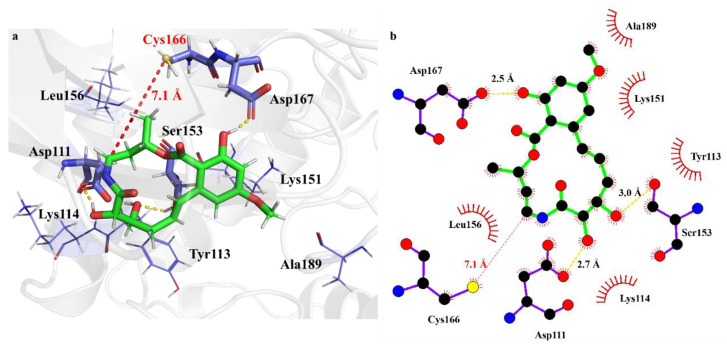
Interaction modes of mod-P toward ERK2 at the non-covalent binding state, (**a**) 3D diagram of the binding pocket, and (**b**) 2D interaction diagram showing the hydrogen bonds and hydrophobic residues. The green sticks represents molecule mod-P, blue sticks represents residues of ERK2 formed interaction with mod-P, yellow dash represents hydrogen bonds.

**Figure 9:**
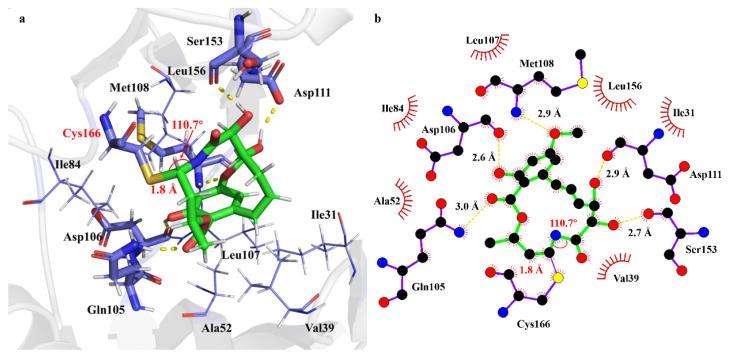
Interaction modes of mod-P toward ERK2 at the covalent binding state, (**a**) 3D diagram of the binding pocket, and (**b**) 2D interaction diagram showing the hydrogen bonds and hydrophobic residues. The green sticks represents molecule mod-P, blue sticks represents residues of ERK2 formed interaction with mod-P, yellow dash represents hydrogen bonds.

The interaction modes of mod-P with ERK2 at noncovalent and covalent binding states are shown in Figure 8 and Figure 9, respectively. For the non-covalent binding, the complex of mod-P with ERK2 involves similar hydrogen bonds as nonPE, but the hydrophobic cavity is different in view of the surrounding residues. The binding free energy of mod-P to ERK2 agrees with that of Ponatinib within the computational errors. Although the electrostatic interaction of mod-P with ERK2 is almost two-folds stronger than that of Ponatinib, the polar-solvation free energy is so high that the binding free energy is compensated considerably. For the covalent binding, the association of mod-P with ERK2 becomes evidently stronger as indicated by the binding free energy of 42 kcal/mol, which is 8.6 kcal/mol higher than that for Ponatinib. In comparison with the covPE complex, the mod-P/ERK2 complex involves more hydrogen bonds, e.g., Asp110 with the 4′-OH and Met108 with the terminal methoxy, where both Asp110 and Met108 residues merge from the hydrophobic region. The new hydrophobic cavity for the mod-P/ERK2 complex includes two new residues, i.e., Ile84 and Leu107, besides the common Ile31, Val39, Ala52, and Leu156. All the above modifications can contribute more or less to the affinity of mod-P with ERK2. In addition, binding pose metadynamics (BMPD) was performed to explore the binding stability of the ligands. The PoseScore values for Ponatinib and mod-P were calculated to be 4.04 and 1.57, respectively (Figure S4 in the Supporting Information). Therefore, mod-P possesses better binding affinity than Ponatinib toward ERK2, which is consistent with the prediction by the binding energy calculations. Moreover, the ContactScore values for Ponatinib and mod-P were estimated to be 0.621 and 0.647, respectively, suggesting that the nonbonding interactions in both covPE and covPE-mod complexes are persistent enough for the stable ligand–protein binding. It is therefore worth testing the potent biological activity of mod-P for the inhibition of ERK2.

## 3. Materials and Methods

### 3.1. Molecular Dynamics Simulation

MD simulations were conducted on the covalently bonded complex of Ponatinib with ERK2 and the regular non-covalent complex, denoted as covPE and nonPE, respectively. For the sake of comparison, MD simulation was performed on the non-covalent complex of FR with ERK2, i.e., nonFE under the identical experimental conditions. On the basis of the experimental structure (PDB: 3W55), Ponatinib was docked into the binding pocket of ERK2 to form a covalent bond with the residue Cys166 by means of a covalent molecular docking approach [34], resulting in the covPE complex. Meanwhile, Ponatinib and FR were docked into the respective binding pockets using the conventional docking technique to generate the corresponding non-covalent complexes nonPE and nonFE. The reliability of docking was verified by re-docking the ligand extracted from the experimental structure into the docking pocket. Subsequently, the restrained electrostatic potential (RESP) charges [35] of the ligands were calculated using the Antechamber module [36] and Gaussian16 programs [37]. All the complexes were simulated using the GAFF2 force field [38] for ligands and the Amberff19SB protein force field [39] for ERK2, as implemented in the LEaP module. The complexes were solvated in a 10.3 nm × 10.3 nm × 10.3 nm cubic box by the TIP3P water molecules with the periodic boundary conditions. The whole systems were neutralized by adding Na^+^ and Cl^-^ ions for charge balance. The non-bonded cutoff distance was set to be 8 Å, and the particle mesh Ewald method was employed to calculate the long-range electrostatic interactions.

The simulation cells were subjected to energy minimization using the steepest descent method for 500 steps, followed by the conjugate gradient method for 4500 steps. Subsequently, the systems were heated slowly to the ambient conditions (e.g., 298.15 K) within 200 ps by the consecutive constant temperature and fixed volume (NVT) simulations. A 500 ps NVT ensemble equilibration simulation was performed to ensure that the solvent molecules are distributed uniformly in the solvent box. Then, the systems were simulated for 500 ps under the constant atmospheric pressure and 298.15 K (NPT). Finally, each of the equilibrated systems was simulated for 100 ns for production. The integration time step was 2 fs, and the trajectory data were saved every 100 ps for analysis. RMSD and E_TOT_ of the complexes were monitored to examine the relative stability of the systems in terms of both structure and energy.

### 3.2. Binding Free Energy

The binding free energy (Δ*G*_bind_) of the non-covalent nonPE and nonFE complexes was calculated using the molecular mechanics/Poisson–Boltzmann surface area (MM/PBSA) method, as implemented in the Ambertools programs [40]. Using the last 40 ns MD trajectories, the binding free energy of the complexes due to the non-covalent interactions can be obtained by Formulas (1) and (2), i.e.,

(1) $${∆G}_{bind}={∆G}_{complex}-({∆G}_{ERK2}+{∆G}_{ligand})$$

(2) $${∆G}_{bind}={∆E}_{int}+{∆E}_{VDW}+{∆E}_{elec}+{∆G}_{PB}+{∆G}_{SA}$$

where Δ*E*_int_ represents the internal potential energy; Δ*E*_VDW_ and Δ*E*_elec_ are the van der Waals and electrostatic interaction energies, respectively; Δ*G*_PB_ and Δ*G*_SA_ correspond to the polar and nonpolar solvation free energies, respectively. As for the covalent complex, the binding free energy was computed using the hybrid QM/MM method as implemented in the ONIOM protocols [41,42]. Briefly, the Cartesian coordinates of the covPE and nonPE complexes from the MD trajectories were used to generate the double-layer ONIOM(QM:MM) model. The high-level QM layer includes Ponatinib and the Cys166 residue of ERK2 as calculated at the M06-2X/def2-TZVP level of theory [43,44]. The low-level MM layer contains all the remaining residues of ERK2 as treated using the Amberff19SB protein force field [39]. The solvent molecules and ions were excluded for simplification. Note that the ONIOM calculations were performed by an electrostatic embedding approach for both optimization and thermodynamic statistical analysis.

### 3.3. Reaction Mechanisms for the Covalent Bonding

The covalent bonding between Ponatinib and ERK2 was simulated using the reaction of Ponatinib with cysteine molecules. Geometries of reactants, transition states, and products were fully optimized using the density functional M06-2X [43] with the standard 6-31G(d,p) basis set [45]. The effect of solvation was included implicitly using the polarizable continuum model (PCM) with the integral equation formalism variant [46]. Harmonic vibrational frequencies were calculated at the same level to obtain the zero-point energy (ZPE) and free-energy corrections and to confirm the nature of stationary point. The minimum has all real frequencies, and the transition state has only one imaginary frequency corresponding to the vibration along the designated reaction coordinate. Intrinsic reaction coordinate calculations [47,48] were employed to verify the connectivity between the transition states and the corresponding reactants and products. All the ab initio calculations were performed using the Gaussian16 programs [37].

### 3.4. Binding Pose Metadynamics

Binding pose metadynamics (BPMD) is an automated enhanced sampling metadynamics-based approach, in which the ligand is forced to move in and around its binding pose. Instead of running long metadynamics simulations until the free-energy surface is fully converged, multiple candidate poses are perturbed in short simulations. These poses are then evaluated by stability using the observed RMSD relative to the initial ligand coordinates and persistence of hydrogen bonds during the metadynamics simulations [49]. The stable coordinates of covPE and covPE-mod were retrieved to conduct BPMD using OpenBPMD, an open-source Python reimplementation and reinterpretation of BPMD [50]. After the potential energy minimization of 10,000 steps, the systems were equilibrated by 500 ps NVT ensemble sampling to prove the system slowly reached the desired temperature of 300 K. For each system, a total of 10 independent metadynamics production simulations of 10 ns were performed using the RMSD of all the heavy atoms from the starting pose as the collective variables (CV). PoseScore, representing the average RMSD from the beginning pose, was calculated to be the indication of the ligand stability during the simulations. In addition, ContactScore, a metric similar to PersScore but taking not only hydrogen bonds but also other nonbonding interactions into consideration, was calculated to track the persistence of the complexation between the ligand and protein [49].

## 4. Conclusions

The mechanisms for the covalent binding of Ponatinib with ERK2 have been clarified in detail on the basis of molecular dynamics simulations and quantum chemistry calculations. The Ponatinib molecule enters the binding pocket of ERK2, which consists of both hydrogen bonds and a hydrophobic cavity. Subsequently, the Cys166 residue of ERK2 approaches the α,β-C=C-unsaturated moiety. The S and H atoms of the thiol group of Cys166 are added to the 8′-C and 7′-C atoms of Ponatinib, respectively, to form the stable adduct with the -C(S)-C(H)- sketch via a four-center barrier or a six-center H_2_O-catalyzed barrier. The binding free energies of the non-covalent and covalent inhibitions are 14.8 kcal/mol and 33.4 kcal/mol, respectively. The barrier height for the covalent binding was estimated to be 47.2 kcal/mol. In case the C=C bonds were saturated, both covalent binding and also spontaneous complexation cannot take place. However, the covalent reaction might be carried out efficiently once the α,β-C=C bond is substituted by the α,β-C=N bond, leading to the -C(S)-N(H)-type complex. The barrier height is reduced to only 23.1 kcal/mol, and the covalent complex becomes even more stable. The modified Ponatinib appears to be a potential inhibitor to ERK2. These findings elucidate new insights into the mechanistic aspects of the covalent bonding and interaction modes between the α,β-unsaturated molecules and ERK2, proving that targeting on the residue Cys166 of ERK2 by the nucleophilicity covalent inhibitor is feasible. The results provide a theoretical basis for designing novel α,β-unsaturated ketone-based ERK2 covalent inhibitors.

## Figures and Tables

**Table 1:** Predicted binding energies (in kcal/mol) of various complexes.

Complexes	Δ*E*_vdw_	Δ*E*_elec_	Δ*G*_PB_	Δ*G*_SA_	Δ*G*_bind_
nonPE	−37.1 ± 3.2	−24.7 ± 7.8	50.7 ± 6.9	−3.6 ± 0.3	−14.8 ± 3.2
covPE					−33.4 ± 3.0
nonFE	−28.4 ± 4.3	−21.1 ± 7.9	75.6 ± 7.3	−2.1 ± 0.6	24.0 ± 4.8
nonPE-mod	−35.7 ± 4.0	−53.4 ± 7.3	82.1 ± 6.2	−3.1 ± 1.3	−12.1 ± 4.7
covPE-mod					−42.0 ± 4.0

**Table 2:** Zero-point energy corrections (ΔZPE) and the relative electronic (Δ*E*) and free energies at 298.15 K for the species involved in the reaction of Ponatinib with cysteine calculated at the M06-2X/6-31G(d,p)-PCM level of theory (units: kcal/mol).

Species	ΔZPE	Δ*E*	Δ*G*
TS1	−1.1	34.2	47.2
P1	3.3	−28.2	−12.1
TS2	−1.5	44.1	56.2
P2	3.7	−27.4	−9.4
TS3	−0.35	40.3	51.7
P3	5.1	−29.1	−8.2
wTS1	2.0	21.2	47.5
wTS2	2.1	29.8	55.7
mod-TS1	0.15	24.1	37.6
mod-P1	4.4	−34.6	−16.3
mod-wTS1	2.5	−5.3	23.1

## Data Availability

Not applicable.

## Supplementary Materials

The supporting information can be downloaded at: https://www.mdpi.com/article/10.3390/ijms242015223/s1.

## References

[1] O’Neill E., Kolch W. (2004). Conferring specificity on the ubiquitous Raf/MEK signalling pathway. Br. J. Cancer..

[2] Kolch W. (2005). Coordinating ERK/MAPK signalling through scaffolds and inhibitors. Nat. Rev. Mol. Cell Biol..

[3] Roberts P.J., Der C.J. (2007). Targeting the Raf-MEK-ERK mitogen-activated protein kinase cascade for the treatment of cancer. Oncogene.

[4] Chen D., Si W., Shen J., Du C., Lou W., Bao C., Zheng H., Pan J., Zhong G., Xu L. (2018). miR-27b-3p inhibits proliferation and potentially reverses multi-chemoresistance by targeting CBLB/GRB2 in breast cancer cells. Cell Death Dis..

[5] Liu S., Zha J., Lei M. (2018). Inhibiting ERK/Mnk/eIF4E broadly sensitizes ovarian cancer response to chemotherapy. Clin. Transl. Oncol..

[6] Ma J., Zeng S., Zhang Y., Deng G., Qu Y., Guo C., Yin L., Han Y., Cai C., Li Y. (2017). BMP4 promotes oxaliplatin resistance by an induction of epithelial-mesenchymal transition via MEK1/ERK/ELK1 signaling in hepatocellular carcinoma. Cancer Lett..

[7] Gagliardi M., Pitner M.K., Park J., Xie X., Saso H., Larson R.A., Sammons R.M., Chen H., Wei C., Masuda H. (2020). Differential functions of ERK1 and ERK2 in lung metastasis processes in triple-negative breast cancer. Sci. Rep..

[8] Chapman P.B., Hauschild A., Robert C., Haanen J.B., Ascierto P., Larkin J., Dummer R., Garbe C., Testori A., Maio M. (2011). Improved survival with vemurafenib in melanoma with BRAF V600E mutation. N. Engl. J. Med..

[9] Long G.V., Trefzer U., Davies M.A., Kefford R.F., Ascierto P.A., Chapman P.B., Puzanov I., Hauschild A., Robert C., Algazi A. (2012). Dabrafenib in patients with Val600Glu or Val600Lys BRAF-mutant melanoma metastatic to the brain (BREAK-MB): A multicentre, open-label, phase 2 trial. Lancet Oncol..

[10] Degirmenci U., Wang M., Hu J. (2020). Targeting aberrant RAS/RAF/MEK/ERK signaling for cancer therapy. Cells.

[11] Hatzivassiliou G., Liu B., O’Brien C., Spoerke J.M., Hoeflich K.P., Haverty P.M., Soriano R., Forrest W.F., Heldens S., Chen H. (2012). ERK Inhibition Overcomes Acquired Resistance to MEK Inhibitors. Mol. Cancer Ther..

[12] Morris E.J., Jha S., Restaino C.R., Dayananth P., Zhu H., Cooper A., Carr D., Deng Y., Jin W., Black S. (2013). Discovery of a novel ERK inhibitor with activity in models of acquired resistance to BRAF and MEK inhibitors. Cancer Discov..

[13] Brenan L., Andreev A., Cohen O., Pantel S., Kamburov A., Cacchiarelli D., Persky N.S., Zhu C., Bagul M., Goetz E.M. (2016). Phenotypic characterization of a comprehensive set of MAPK1/ERK2 missense mutants. Cell Rep..

[14] Roskoski R. (2019). Targeting ERK1/2 protein-serine/threonine kinases in human cancers. Pharmacol. Res..

[15] Blake J.F., Burkard M., Chan J., Chen H., Chou K.J., Diaz D., Dudley D.A., Gaudino J.J., Gould S.E., Grina J. (2016). Discovery of (S)-1-(1-(4-Chloro-3-fluorophenyl)-2-hydroxyethyl)-4-(2-((1-methyl-1 H-pyrazol-5-yl) amino) pyrimidin-4-yl) pyridin-2 (1 H)-one (GDC-0994), an Extracellular Signal-Regulated Kinase 1/2 (ERK1/2). J. Med. Chem..

[16] Wong D.J., Robert L., Atefi M.S., Lassen A., Avarappatt G., Cerniglia M., Avramis E., Tsoi J., Foulad D., Graeber T.G. (2014). Antitumor activity of the ERK inhibitor SCH722984 against BRAF mutant, NRAS mutant and wild-type melanoma. Mol. Cancer..

[17] St Denis J.D., Chessari G., Cleasby A., Cons B.D., Cowan S., Dalton S.E., East C., Murray C.W., O’Reilly M., Peakman T. (2022). X-ray screening of an electrophilic fragment library and application toward the development of a novel ERK 1/2 covalent inhibitor. J. Med. Chem..

[18] Chiang C.-Y., Zhang M., Huang J., Zeng J., Chen C., Pan D., Yang H., Zhang T., Yang M., Han Q. (2023). A Novel Selective ERK1/2 Inhibitor, Laxiflorin B, Targets EGFR Mutation Subtypes in Non-small-cell Lung Cancer. Acta Pharmacol. Sin..

[19] Miller V.A., Hirsh V., Cadranel J., Chen Y.-M., Park K., Kim S.-W., Zhou C., Su W.-C., Wang M., Sun Y. (2012). Afatinib versus placebo for patients with advanced, metastatic non-small-cell lung cancer after failure of erlotinib, gefitinib, or both, and one or two lines of chemotherapy (LUX-Lung 1): A phase 2b/3 randomised trial. Lancet Oncol..

[20] Wu Y.-L., Cheng Y., Zhou X., Lee K.H., Nakagawa K., Niho S., Tsuji F., Linke R., Rosell R., Corral J. (2017). Dacomitinib versus Gefitinib as First-Line Treatment for Patients with EGFR-Mutation-Positive Non-Small-Cell Lung Cancer (ARCHER 1050): A Randomised, Open-Label, Phase 3 Trial. Lancet Oncol..

[21] Tam C.S., Opat S., D’Sa S., Jurczak W., Lee H.-P., Cull G., Owen R.G., Marlton P., Wahlin B.E., Sanz R.G. (2020). A Randomized Phase 3 Trial of Zanubrutinib vs Ibrutinib in Symptomatic Waldenström Macroglobulinemia: The ASPEN Study. Blood.

[22] Chen R., Wang Z., Liu L., Pan Z. (2022). Discovery of novel photocaged ERK1/2 inhibitors as light-controlled anticancer agents. Chem. Commun..

[23] Pan X., Pei J., Wang A., Shuai W., Feng L., Bu F., Zhu Y., Zhang L., Wang G. (2022). Development of small molecule extracellular signal-regulated kinases (ERKs) inhibitors for cancer therapy. Acta Pharm. Sin. B.

[24] Bauer R.A. (2015). Covalent inhibitors in drug discovery: From accidental discoveries to avoided liabilities and designed therapies. Drug Discov. Today.

[25] Ghosh A.K., Samanta I., Mondal A., Liu W.R. (2019). Covalent inhibition in drug discovery. ChemMedChem.

[26] Roth G.J., Stanford N., Majerus P.W. (1975). X Acetylation of prostaglandin synthase by aspirin. Proc. Natl. Acad. Sci. USA.

[27] Smith J.B., Willis A.L. (1971). Aspirin selectively inhibits prostaglandin production in human platelets. Nature New Biol..

[28] Hammond J., Leister-Tebbe H., Gardner A., Abreu P., Bao W., Wisemandle W., Baniecki M., Hendrick V.M., Damle B., Simón-Campos A. (2022). Oral nirmatrelvir for high-risk, nonhospitalized adults with Covid-19. N. Engl. J. Med..

[29] Marzolini C., Kuritzkes D.R., Marra F., Boyle A., Gibbons S., Flexner C., Pozniak A., Boffito M., Waters L., Burger D. (2022). Recommendations for the management of drug–drug interactions between the COVID-19 antiviral nirmatrelvir/ritonavir (Paxlovid) and comedications. Clin. Pharmacol. Ther..

[30] Singh J., Petter R.C., Baillie T.A., Whitty A. (2011). The resurgence of covalent drugs. Nat. Rev. Drug Discov..

[31] Boike L., Henning N.J., Nomura D.K. (2022). Advances in covalent drug discovery. Nat. Rev. Drug Discov..

[32] Mah R., Thomas J.R., Shafer C.M. (2014). Drug discovery considerations in the development of covalent inhibitors. Bioorg. Med. Chem. Lett..

[33] Ohori M., Kinoshita T., Yoshimura S., Warizaya M., Nakajima H., Miyake H. (2007). Role of a cysteine residue in the active site of ERK and the MAPKK family. Biochem. Biophys. Res. Commun..

[34] Bianco G., Forli S., Goodsell D.S., Olson A.J. (2016). Covalent docking using autodock: Two-point attractor and flexible side chain methods. Protein Sci..

[35] Schauperl M., Nerenberg P.S., Jang H., Wang L.-P., Bayly C.I., Mobley D.L., Gilson M.K. (2020). Non-bonded force field model with advanced restrained electrostatic potential charges (RESP2). Commun. Chem..

[36] Wang J., Wang W., Kollman P.A., Case D.A. (2001). Antechamber: An accessory software package for molecular mechanical calculations. Abstr. Pap. Am. Chem. Soc..

[37] Frisch M.J., Trucks G.W., Schlegel H.B., Scuseria G.E., Robb M.A., Cheeseman J.R., Scalmani G., Barone V., Petersson G.A., Nakatsuji H. (2016). Gaussian 16, Revision C.01.

[38] Wang J., Wolf R.M., Caldwell J.W., Kollman P.A., Case D.A. (2004). Development and testing of a general amber force field. J. Comput. Chem..

[39] Tian C., Kasavajhala K., Belfon K.A.A., Raguette L., Huang H., Migues A.N., Bickel J., Wang Y., Pincay J., Wu Q. (2019). ff19SB: Amino-acid-specific protein backbone parameters trained against quantum mechanics energy surfaces in solution. J. Chem. Theory Comput..

[40] Miller III B.R., McGee T.D., Swails J.M., Homeyer N., Gohlke H., Roitberg A.E. (2012). MMPBSA. py: An efficient program for end-state free energy calculations. J. Chem. Theory Comput..

[41] Chung L.W., Sameera W.M.C., Ramozzi R., Page A.J., Hatanaka M., Petrova G.P., Harris T.V., Li X., Ke Z., Liu F. (2015). The ONIOM method and its applications. Chem. Rev..

[42] Awoonor-Williams E., Abu-Saleh A.A.A.A. (2021). Covalent and non-covalent binding free energy calculations for peptidomimetic inhibitors of SARS-CoV-2 main protease. Phys. Chem. Chem. Phys..

[43] Zhao Y., Truhlar D.G. (2008). The M06 suite of density functionals for main group thermochemistry, thermochemical kinetics, noncovalent interactions, excited states, and transition elements: Two new functionals and systematic testing of four M06-class functionals and 12 other functionals. Theor. Chem. Acc..

[44] Weigend F., Ahlrichs R. (2005). Balanced basis sets of split valence, triple zeta valence and quadruple zeta valence quality for H to Rn: Design and assessment of accuracy. Phys. Chem. Chem. Phys..

[45] Ditchfield R.H.W.J., Hehre W.J., Pople J.A. (1971). Self-consistent molecular-orbital methods. IX. An extended Gaussian-type basis for molecular-orbital studies of organic molecules. J. Chem. Phys..

[46] Miertuš S., Scrocco E., Tomasi J. (1981). Electrostatic interaction of a solute with a continuum. A direct utilizaion of AB initio molecular potentials for the prevision of solvent effects. Chem. Phys..

[47] Fukui K. (1970). Formulation of the reaction coordinate. J. Phys. Chem..

[48] Fukui K. (1981). The path of chemical reactions-the IRC approach. Acc. Chem. Res..

[49] Fusani L., Palmer D.S., Somers D.O., Wall I.D. (2020). Exploring ligand stability in protein crystal structures using binding pose metadynamics. J. Chem. Inf. Model..

[50] Lukauskis D., Samways M.L., Aureli S., Cossins B.P., Taylor R.D., Gervasio F.L. (2022). Open Binding Pose Metadynamics: An Effective Approach for the Ranking of Protein–Ligand Binding Poses. J. Chem. Inf. Model..

