# Supplementary material for: Computational Investigations on Reaction Mechanisms of the Covalent Inhibitors Ponatinib and Analogs Targeting the Extracellular Signal-Regulated Kinases
Source: Int J Mol Sci. 2023 Oct 16;24(20):15223. doi: 10.3390/ijms242015223 (PMC10607051; doi:10.3390/ijms242015223)
Supplement: Supplementary file 1 [file ijms-24-15223-s001.zip › ijms-2642557-supplementary.pdf]

Supporting Information

Computational Investigations on Reaction Mechanisms of the Covalent Inhibitors Ponatinib and Analogs Targeting the Extracellular Signal-regulated Kinases

TIAN Yafeng, ZHANG Mi, HENG Panpan, HOU Hua, WANG Baoshan*
(College of Chemistry and Molecular Sciences, Wuhan University, Wuhan 430072, China)

*Corresponding author. E-mail: baoshan@whu.edu.cn

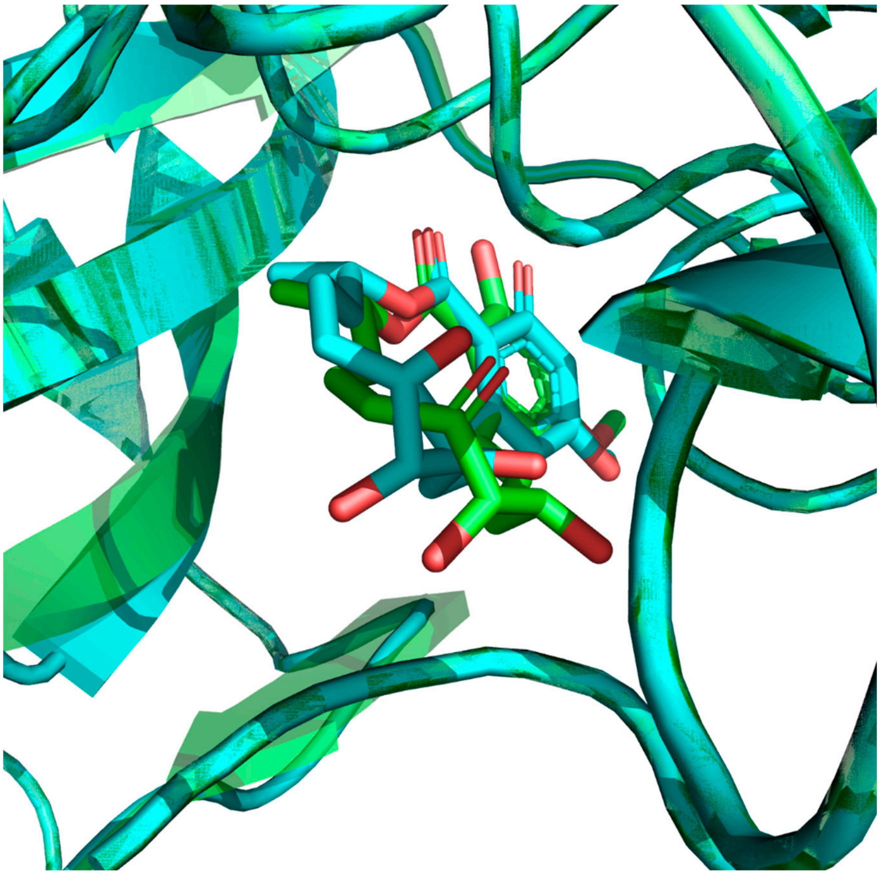

Figure S1. Re-docked co-crystal ligand/ERK2 in the binding pocket. Green sticks: Docked ligand. Cyan sticks: Experimental ligand.

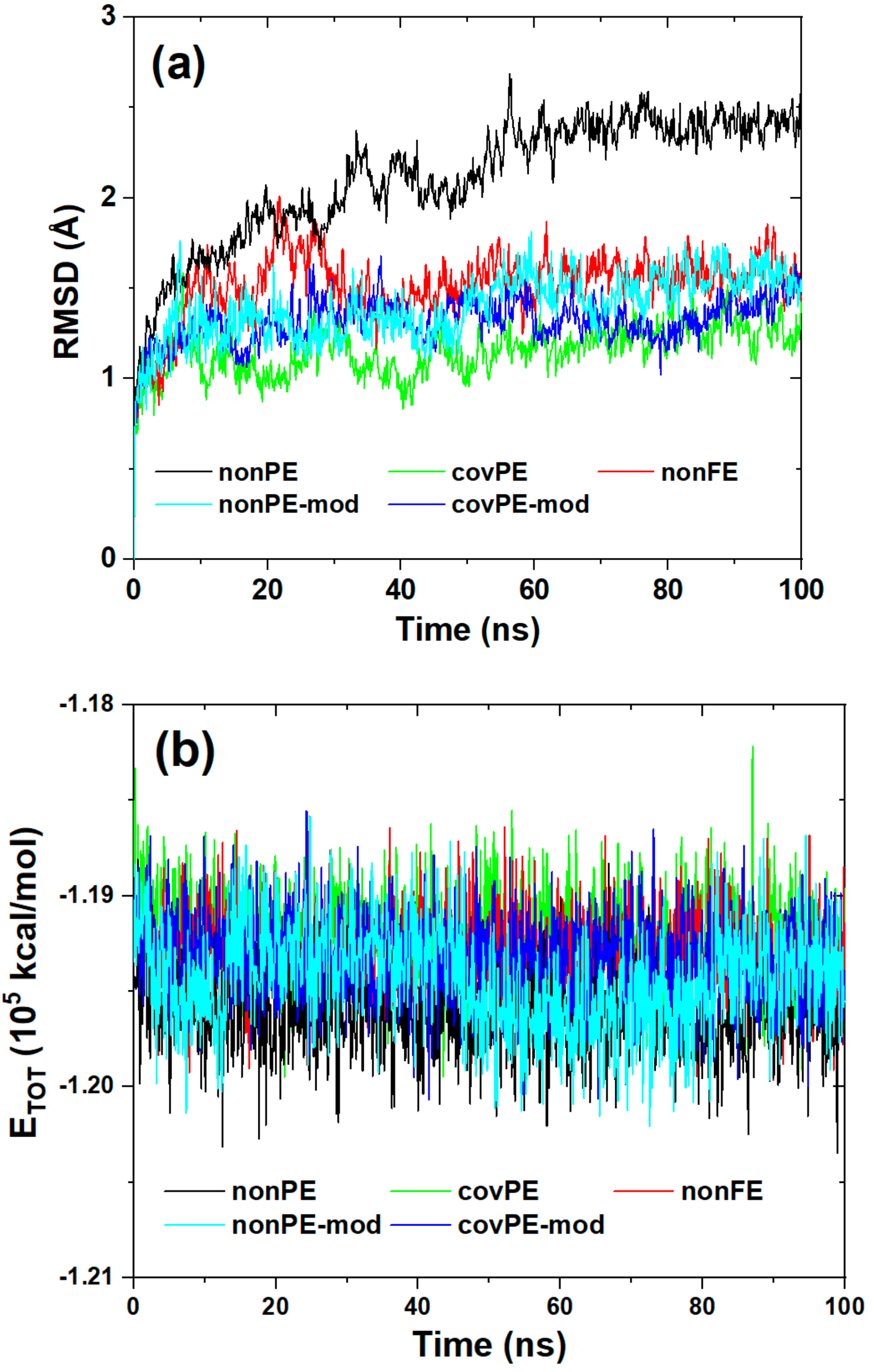

Figure S2. Temporal profiles for the RMSD of the backbone atoms of the protein (a) and the E_{TOT} (b) data for various inhibitor-ERK2 complexes.

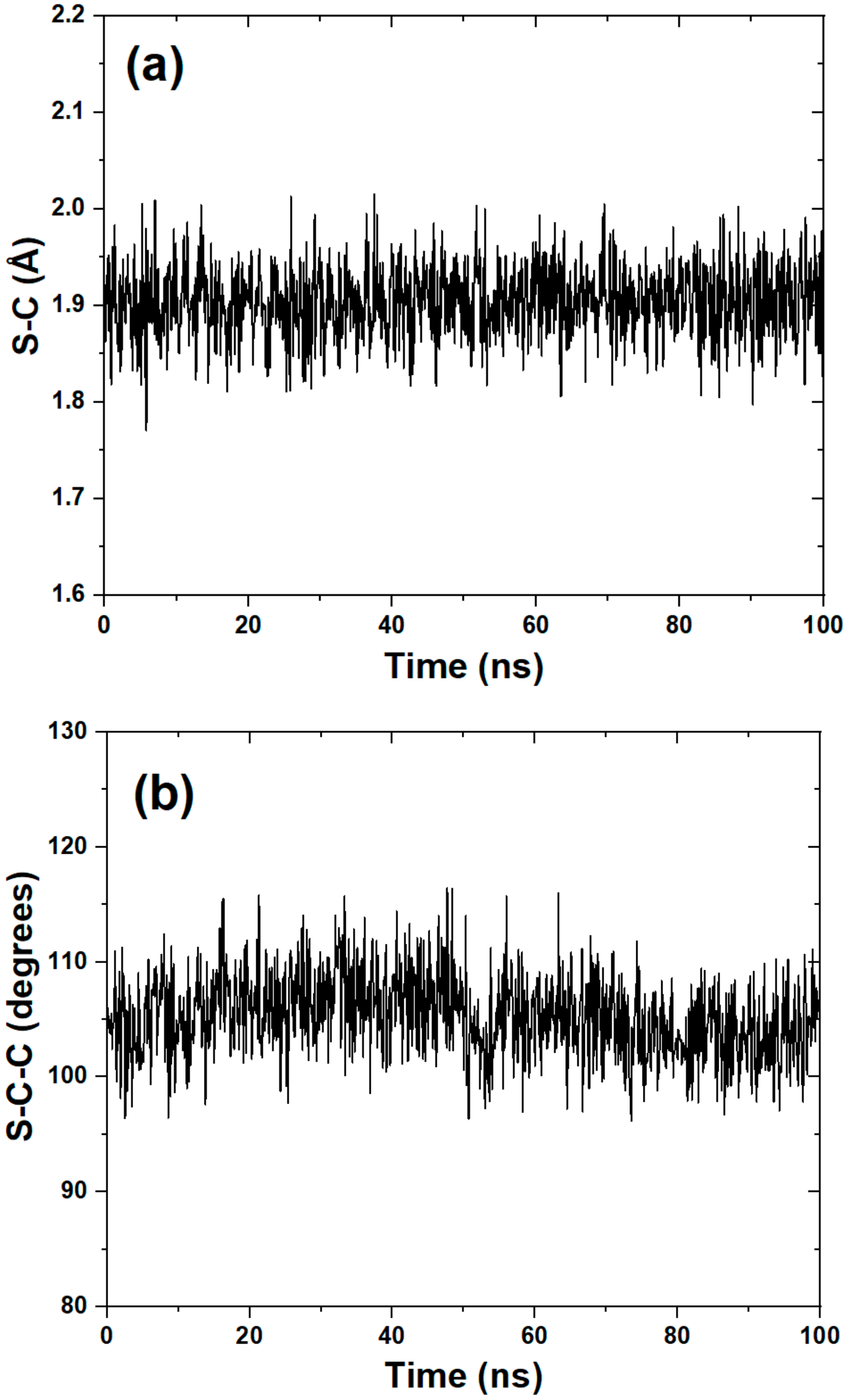

Figure S3. Temporal profiles for the characteristic geometrical parameters of the covalent bond between Ponatinib and the Cys166 residue: (a) S-C bond distances. (b) S-C-C bond angles.

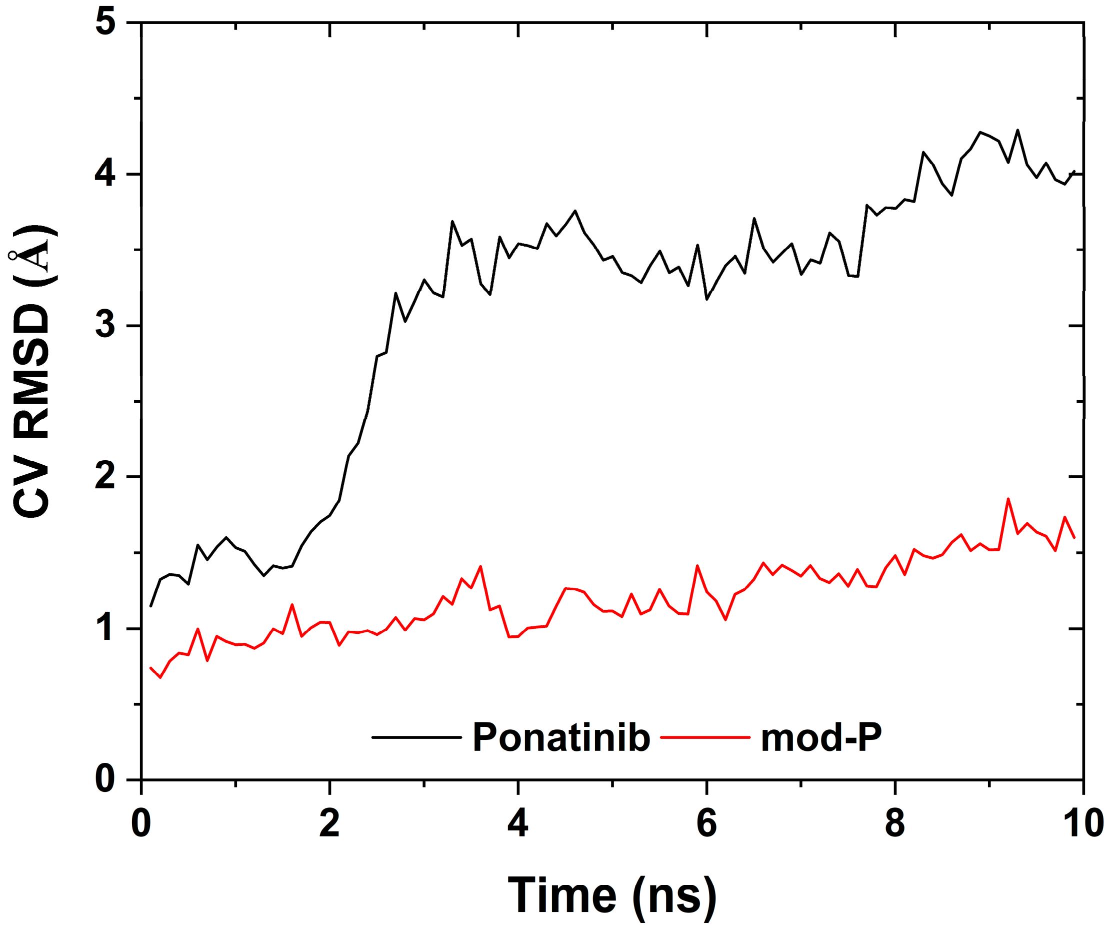

Figure S4. Average CV RMSD of Ponatinib and mod-P conformation over 10 × 10 ns metadynamics runs.
